# Factors Contributing to Successful Spontaneous Dog–Human Cooperation

**DOI:** 10.3390/ani13142390

**Published:** 2023-07-23

**Authors:** Melitta Csepregi, Márta Gácsi

**Affiliations:** 1 ELKH–ELTE Comparative Ethology Research Group, 1117 Budapest, Hungary; marta.gacsi@ttk.elte.hu; 2 Department of Ethology, Eötvös Loránd University, 1117 Budapest, Hungary; 3 Doctoral School of Biology, Institute of Biology, Eötvös Loránd University, 1117 Budapest, Hungary

**Keywords:** dog, cooperation, dog–human interaction

## Abstract

**Simple Summary:**

Dogs are undeniably capable of effective cooperation with humans, whether their task is herding, hunting, or assisting disabled people. However, the factors influencing family dogs’ spontaneous tendency to cooperate remains unexplored. Our aim was to investigate if breed function, training experience, and owner-reported social motivation level were associated with successful spontaneous dog–owner cooperation. We tested family dogs from different breed groups (non-working dogs, cooperative/independent working breeds), with various training backgrounds in a cooperative task, in which they had to work together with their owners to achieve a common goal. We measured whether dogs paid attention to their partner, understood the problem situation, and were willing to help their owners. Breed groups had no significant effect on the dogs’ behaviour. Dogs with high training levels and high social motivation were more attentive and more cooperative. This implies that in future studies, special attention should be dedicated to consider the subjects’ training background and social motivation. Our findings emphasise the need for test procedures that do not rely on specific trained skills of the dogs.

**Abstract:**

Dogs’ ability to cooperate with humans is widely acknowledged, but the factors influencing their spontaneous cooperative tendencies are largely unknown. We investigated whether breed function, training experience, and owner-reported social motivation level contribute to spontaneous dog–owner cooperation. Family dogs (N = 100) of three breed groups (non-working dogs, cooperative/independent working breeds) with various training experiences were tested in an ‘out–of–reach’ task with their owners as their partners, who never directly asked for help during the test. We measured dogs’ behaviour along three main components of successful cooperation: paying attention, understanding the problem, and willingness to cooperate. Breed groups had no significant effect on dogs’ behaviour. No factor was associated with the behavioural variables related to not understanding the task. Dogs with high training levels and high social motivation showed more attention-related behaviours and were more likely to help the owner (training level and social motivation were not correlated with each other). Our results highlight the importance of training experience and social motivation in dogs’ attentiveness and spontaneous cooperativity. This also points to the need for careful sample balancing and experimental procedures that do not rely on specific trained skills.

## 1. Introduction

Cooperative behaviours are joint, synchronised, and sometimes even complementary actions performed by two or more individuals, that benefit at least one of them in a way that could not have been achieved individually [1]. When it comes to cooperation, dogs hold a special position. They often engage in cooperative interactions with humans, such as assisting disabled people, hunting, or herding, which establishes their significance as research subjects in their own right [2]. Although most forms of dog–human cooperation are based on training to a certain degree, considering dogs’ potential to engage in various interspecific interactions with humans compared to, for example, highly socialised wolves [3], we argue that the aforementioned behaviours cannot be solely attributed to training. For example, Naderi et al. [2] reported that naive pet dogs showed a surprisingly successful performance when they had to help their owner through an obstacle course, although they ‘showed a less professional performance’ when compared to trained guide dogs. Their remarkable parallels with humans in certain important skills (e.g., social learning [4], relying on pointing signals [5] even as early as 6–8 weeks of age [6,7]) and behaviours (e.g., attachment: [8,9,10]) make them valuable model animals to study the evolutionary aspects of human cooperativity. In addition, the study of interspecific communication and cooperation between dogs and humans carries the potential to serve as a model in the rapidly expanding field of social robotics, facilitating more natural and effective interactions between robots and humans [11].

Dogs engage in cooperative interactions both with other dogs and with humans. To date, there are limited data available regarding dogs’ cooperativity with conspecifics, and the available findings present somewhat contradicting results. In a prosocial bar-pulling task, pet dogs were reported to provide food to another dog only if the partner was familiar to them [12]. Gfrerer and Taborsky [13] used an iterated prisoner’s dilemma paradigm to test military service dogs (all Malinois). The subjects of this study reciprocated help after receiving help themselves, regardless of whether the other dog was a cooperative partner or an unknown one (seemingly following the ‘help anyone if helped by someone’ rule). However, [14] reported that dogs did not act prosocially in a touch screen task, in which they could provide food to in-group or out-group conspecifics by selecting a specific symbol. Knowledge-probe trials were also included after the test sessions to test dogs’ motivation and understanding of the task: in these trials, subjects themselves received the food rewards. Dogs resumed working in these trials, suggesting that their absence of prosocial behaviours was not due to a lack of understanding of the task.

Cooperation is perhaps even more substantial in dog–human interactions. It has been proposed that dogs and humans underwent a convergent evolution due to the similar environmental factors they were exposed to, and that during dogs’ domestication, immense selective pressure affected their skills in communication and cooperation with humans [15]. This underlies their importance as model animals in the research of the evolutionary processes that shaped human cognition and social behaviour. Furthermore, there is usually no typical dominance hierarchy between dogs and humans [16]. The main reason for this could be that the pet dog–human relationship is asymmetrical: dogs are highly dependent on their owners, who supply them with food, have control over their reproduction, and provide them security [17]. Hence, cooperative behaviours are likely to be far more adaptive in dog–human interactions than competitive ones. Many breeds and breed lines have been specifically selected for engaging in complex cooperative behaviours with humans, such as hunting, herding, or assisting disabled people. Taken together, dog–human cooperation can provide new insights into the evolution of cooperative behaviours. Since cooperativity occurs so frequently in dog–human interactions, sometimes even spontaneously, it is important to gain adequate knowledge about the contexts and conditions among which these behaviours appear.

Investigating the cooperative behaviours of non-human species presents a unique challenge, as observing the subject species in their natural habitat can often prove to be quite strenuous or even impossible. The alternative is to study their behaviour under controlled laboratory conditions, which in turn may not accurately reflect their natural behaviour. Current research on the contexts in which dogs behave cooperatively with humans is primarily based on profoundly artificial laboratory settings, which hardly reflect this species’ natural behaviour (e.g., bar–pulling: [12]; button pushing: [18]). Moreover, laboratory experiments often require extensive pre-training, making it particularly difficult to rule out the effect of learning and to assess the subjects’ spontaneous tendency to cooperate. Adapting the same experimental paradigms from primates to other non-human species can also be challenging, as tasks must often be simplified to match the subject species’ cognitive abilities and/or anatomy (as seen in, e.g., [12]), since primates often manipulate objects with their hands. Thus, we must weigh the advantages and disadvantages of different methodologies and make trade-offs when designing methodologies for the chosen condition, whether it is the species’ natural environment or the laboratory.

Companion dogs’ natural environment is the human niche; therefore, humans became a fundamental part of their social environment [19]. This underlies the relevance and prominence of dog–human cooperation; however, the available literature is quite limited on this topic. Pet dogs helped both their owners and a stranger by pressing a button to enter a locked room, but only if the human partner made their goal ‘as obvious as possible’, e.g., by directly pointing at the button [18]. It has to be mentioned that in this study, dogs were significantly more likely to open the door when there was food on the other side, instead of an object that was only important for the human. Kaminski et al. [20] reported that dogs showed the location of a hidden object more frequently when they were interested in the object themselves (e.g., their favourite toy), compared to when it was solely relevant to their owner (e.g., a pair of scissors). Interestingly, however, dogs did not ‘give up’; owners found an object equally as often in the first and second half of the trials. This suggests that dogs are highly motivated to try to help their human partner, even if they did not understand what exactly the owner needed. According to [21], dogs recruited human partners to perform a string-pulling task with them, and they did so significantly more often in those trials when the task could not be solved without the help of a partner.

The partly contradictory results are not surprising, as the experimental contexts used and the behaviours expected of the dogs were often unusual and did not fit into their natural behaviour repertoire (e.g., opening a door by pushing a button, moving a tray by pulling a string). Therefore, here, we used the ‘out–of–reach’ paradigm with a fairly natural context for the dogs, which did not require prior training to be solved.

We assessed dogs’ behaviours based on the three main components of successful cooperation: (1) paying attention to the partner, (2) understanding the problem situation, and (3) willingness to help the partner in achieving a common goal. We investigated which of the following factors play a role in the success of spontaneous dog–owner cooperation.

(I) Breed function. Although the attachment scores of dog breeds selected for different degrees of cooperation did not differ [22], breeds selected specifically for cooperating with humans outperformed independently working breeds and mongrels in a two-way choice test, implying that they rely more on human communicational gestures [23]. Cooperative and mixed breeds established eye contact with a human faster than non-cooperative breeds [24], which is an important cue of attentiveness and likely improves the effectiveness of dog–human cooperation.

(II) Training experience. Assistance and therapy dogs were reported to be better in independent problem solving compared to untrained dogs and dogs trained for recreational/sporting purposes in a task, where they had to manipulate an apparatus with food hidden inside. Training in general was associated with more problem-oriented behaviour in this [25].

(III) Social motivation. Tomasello et al. [26] argued that humans have a unique motivation to share the perceptions, intentions, and goals of others. This motivation contributes to our ability to engage in intricate forms of cooperativity. Similarly, dogs have been found to recognise others’ goals and act accordingly when they could socially learn various actions from a human demonstrator [27]. Furthermore, in certain training situations, social learning has been found to be more effective than conditioning in pet dogs [28].

We had three separate hypotheses for the aforementioned components. First, we expected that highly trained dogs, dogs with high social motivation (i.e., highly motivated by social rewards, such as praising or petting), and cooperative breeds would be more likely to pay attention to their owners. Second, we expected that if dogs do not understand the problem situation, highly trained and socially motivated individuals would be more prone to show distress responses to the owner’s behaviour. This would suggest that they recognise that the owner has a problem and they are interested in it, but they do not understand the problem situation itself. Third, we expected highly trained dogs and dogs with high social motivation to be more successful in spontaneously cooperating with the owner.

## 2. Materials and Methods

### 2.1. Ethical Statement

Prior to the experiment, participants were informed about the goals and circumstances of the study and signed an informed consent form. Participation was voluntary, and the data obtained were used solely for scientific purposes. Owners had the chance to stop participating at any time. Data collection was approved by the United Ethical Review Committee for Research in Psychology (Permission 2023–04) and the Hungarian Animal Experiments Scientific and Ethical Committee (PE/EA/00035-4/2023). Personally identifiable data were treated confidentially and stored separately from the rest of research data in accordance with applicable data protection laws.

### 2.2. Subjects

We sent out online invitations to owners who had previously participated in other tests at the Department of Ethology with their dogs and advertised our experiment in various Facebook groups to recruit subjects. We tested N = 103 family and assistance dogs, but had to exclude three dogs because their owners did not properly follow the experimental protocol. N = 100 dogs were included in the analysis (mean age ± SD = 5.5 ± 3.2 years, range: 1.1–14.5 years; 41 female [36 neutered], 59 male [49 neutered]). We categorised the dogs based on their function: non-working dogs (9 individuals from 7 breeds and 20 mongrels), independent working breeds (26 individuals from 13 breeds), cooperative working breeds (45 individuals from 19 breeds). Sex and age were counterbalanced as much as possible across breed groups. Basic information about the subjects can be seen in Table 1. For more detailed information about the subjects, see Supplementary Table S1 in the Supplementary material.

Training levels were defined as follows: dogs with basic training were only trained by the owner, or only participated in obedience training. Dogs with advanced training participated in agility, mantrailing, rescuing, defence, and/or hunting training for more than 3 months. The assistance/therapy group consisted of certified assistance and therapy dogs.

### 2.3. Questionnaires

Owners completed an online questionnaire including basic demographic questions about the owners and their dogs, and we assessed (1) dogs’ experience with fetching toys and other objects, (2) dogs’ training experience, and (3) how easily dogs are motivated by social rewards (petting, praising). Based on owner responses for the last question about dogs’ motivation, we established two categories. Dogs that cannot be motivated by social rewards at all, or only for a short period of time, were referred to as the low-social-motivation group. Dogs that were highly motivated by social rewards over a prolonged period of time were referred to as the high-social-motivation group. The questionnaire was incomplete in the case of 6 dogs; therefore, they were not included in every analysis. For the questionnaire, see Section S1 in the Supplementary material.

### 2.4. Procedure

**Figure 1:**
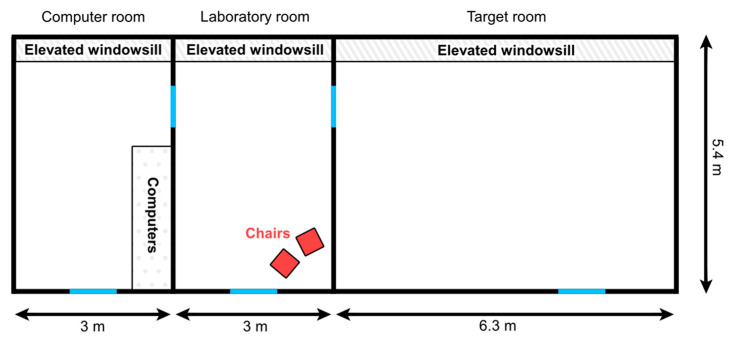
Illustration of the rooms where the experiment was carried out. The blue lines represent doors.

The test was carried out in a laboratory room (3 × 5.4 m) with two adjacent rooms (computer room and target room; see Figure 1 for details) at the Department of Ethology, Eötvös Loránd University. Two light plastic chairs were placed loosely next to each other in the corner of the laboratory room, allowing dogs to push them aside and move in between them if they could not fit under them.

The dog (D) was given 5 min to explore the laboratory room while a female experimenter (E) explained the experimental setup and the protocol to the owner (O). Then, E brought in a bowl of cheese and called O and D with her to the target room, where she showed the contents of the bowl to both of them. (Before each experiment, E made sure that the subject was willing to accept the cheese as a reward.) Afterwards, E placed the bowl on the elevated windowsill and called O and D back to the laboratory room. Then, E locked the door of the target room and placed the key on the chair without attempting to get D’s attention.

A large leather keychain (3.5 × 6 cm) was put on the keyring, which could be picked up easily by D. O was asked to always grab the key (and not the keychain) so that the smell/taste of the cheese did not smear on the leather. (The leather was less easy to clean than the metal key, especially since dogs were more willing to take the keychain in their mouths rather than the key.)

O was provided with Bluetooth headphones that allowed E to give instructions from the computer room during the test. E then left to the computer room to monitor the subjects. Meanwhile, O stood in the centre of the laboratory room, waiting for further instructions from E, while ignoring D completely.

Warm-Up Phase

The objective of this phase was to familiarise the dog with the experimental setting and the significance of the key in gaining access to the target room. During this phase, three trials were conducted, each of which was almost identical.

As per E’s instructions, O retrieved the key from the chair, went to the door leading to the target room, and ‘accidentally’ dropped the key onto the floor while ignoring D. This action gave an emphasis to O’s need for the key, as he/she only attempted to open the door if he/she was in possession of it. The dropping of the key was also important in catching the dogs’ attention.

After picking up the key, O unlocked the door and entered the target room with D. If D did not follow O spontaneously, O called the dog. Depending on the number of the trial, either O, D, or both of them were given a small amount of food in the target room. O was also allowed to pet and praise the dog. Afterwards, O and D went back to the laboratory room together, where O locked the door and placed the key on the chair. They then waited for further instructions from E.

The only difference between the three trials was that in the first trial, both O and D received a small quantity of food; in the second trial, only O received food; and in the third trial, only D received food. Verbal praising and petting were allowed every trial.

Testing Phase

O ‘accidentally’ dropped the key behind the chairs in the corner. As soon as the key fell, E started to measure the time with a stopwatch and continuously instructed O through the entire phase. For the first 15 s, O was instructed to gaze on the key and say fixed phrases, ‘Oh no, I dropped it!’ and ‘I can’t reach it!’, without calling D’s name or using any known words. In the next 15 s, O was instructed to slightly bend down and use reaching (but not pointing gestures) towards the key, while continuing to gaze at the key and repeat the previous sentences. As we intended to investigate dogs’ spontaneous behaviour, O was not allowed to communicate with the dog during the entire trial.

If D did not pick up the key within the first 30 s, O was instructed to attempt to open the locked door of the target room again (e.g., slightly shaking the doorknob), then return to the chairs and continue with the same actions as before (gazing, talking, reaching) for another 15 s. O could repeat this process one more time afterwards.

If D obtained the key from underneath the chair (by pawing or mouthing) and made it accessible to the owner, the trial immediately ended. O was instructed to pick it up and access the food in the target room together with D. O and D both received some food in the target room, and O was allowed to pet and verbally praise D. If D did not manage to obtain the key, the testing phase ended after approximately 1.5 min (2 × 15 s + door opening + 15 s + door opening + 15 s). In this case, the dyad did not receive any rewards.

Test sessions were videotaped and the recordings were coded later with BORIS v.7.13.4 [29].

### 2.5. Behavioural Variables

We measured dogs’ behaviour in relation to the three main components of effective cooperation: (1) paying attention to the partner, (2) comprehending the problem situation (distress response to the owner’s behaviour in case of difficulty in this respect), and (3) cooperative action. Table 2 outlines the behavioural variables associated with each component, accompanied by their definitions.

Due to the slightly varying duration of the experiment among subjects, we used time percentages and calculated the relative frequency of gaze alternations instead of the total number of gaze alternations in the analyses. Behaviours that did not occur frequently enough to be treated as time percentages (vocalising, door) were transformed into binary variables. Although paddling was also coded as a distress response, it occurred so rarely that we decided to exclude it from the analyses altogether.

### 2.6. Statistical Analysis

The inter-rater reliability was assessed on a subsample, and a second coder coded 20% of the subjects. The inter-rater reliability of the variables was tested by calculating Cohen’s kappas, which revealed strong reliability (M ± SD = 0.857 ± 0.214; [30]).

Statistical analyses were performed with R (version 4.2.2). The continuous behavioural variables were transformed with the Box–Cox transformation (‘MASS’ package), if they differed from a normal distribution (Shapiro–Wilk test). None of our independent variables correlated with each other, except for training level and fetching experience.

We used multimodel inference (inference based on the full set of models [31]) to find out which GLMs fitted our data best (‘glmulti’ package). We generated all possible models for every behavioural variable, then assessed the models in terms of AICc (small-sample-corrected Akaike Information Criterion). We selected the best model and the models that were less than 2 IC units away from the best model for further assessment.

Since the AICc values of our models were close to each other, after using the glmulti function, we plotted the relative importance of model terms (i.e., the overall support for each variable across all models [32]). The selection of the final model for each behavioural variable was made based on the following criteria: (1) the model had one of the lowest AICc values, and (2) its terms reached a model-averaged importance of 0.8 or higher. Out of the models within two deltaAICc, we selected the model with the fewest variables.

To model orientation towards owner, closeness to owner, and gaze alternation, Gaussian GLMs were used. Training level, breed group, and social motivation were included in the model, along with all pairwise interaction terms. We used a binomial GLM to model the door variable as a function of the above terms.

We used a binomial GLM to model the occurrence of vocalisations as a function of training level and social motivation, and their pairwise interaction.

To model the approaching key and manipulating key variables, we used two separate binomial GLMs in each variable’s case, as the training level and fetching experience were correlated with each other. The first model included the breed group, training level, social motivation, and the their pairwise interaction terms. The second model included the breed group, fetching experience, social motivation, and their pairwise interactions.

After choosing our final model based on the AICc values and the relative importance of model terms, we used the Tukey test with Benjamini–Hochberg adjustment for post hoc pairwise comparisons between factors (‘emmeans’ package).

For a more comprehensive overview of our results, the models, which were less than 2 IC units away from the best model, and the final models are listed in Section S2 of the Supplementary material for each behavioural variable.

## 3. Results

**Figure 2:**
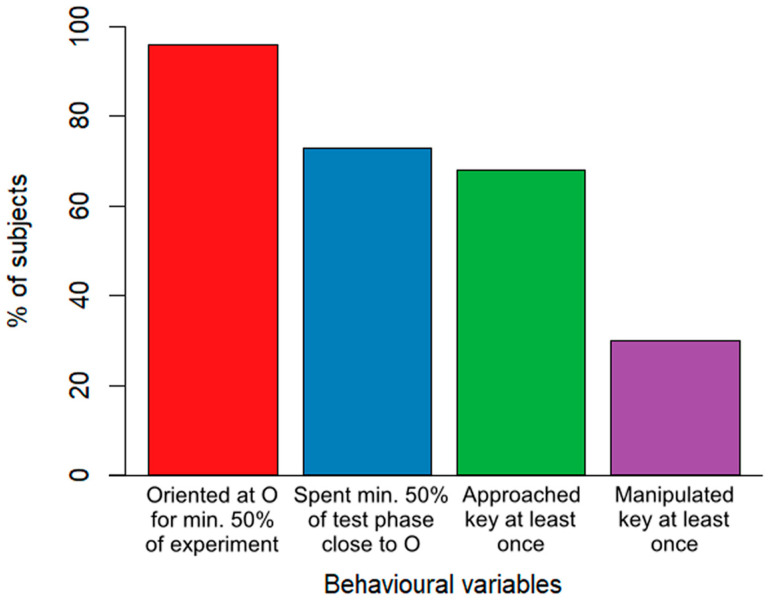
A descriptive breakdown of the percentage of all (N = 100) dogs showing attention and cooperation-related behaviours.

Overall, the majority of dogs showed a high level of attentiveness throughout the experiment. Nearly all of the subjects (96%) oriented towards the owner for at least half of the experiment’s duration. A total of 73% of the subjects remained in close proximity to the owner for at least half of the test phase. Cooperativity-related behaviours were slightly less common, where 68% of the subjects approached the key during the test phase at least once and 30% manipulated the key at least once (Figure 2).

**Figure 3:**
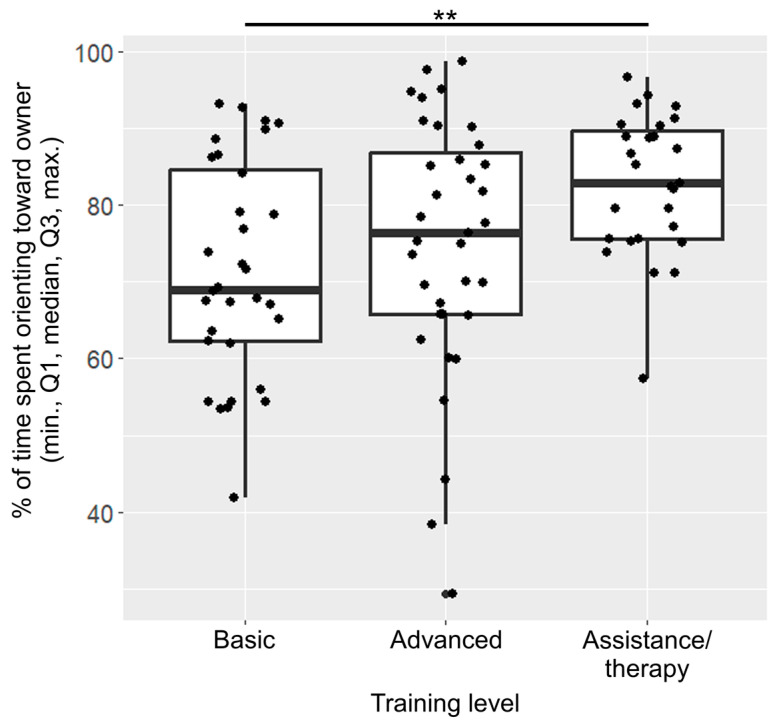
Percentage of time spent orienting towards the owner across the different training levels. The dots represent individual data points. (**: *p* < 0.01).

After the model selection process, only training level was included in the final model of orientation (Figure 3). Dogs with basic training spent a significantly lower percentage of time orienting towards the owner than assistance/therapy dogs (β ± SE  = −6017 ± 1903, *p* = 0.006).

**Figure 4:**
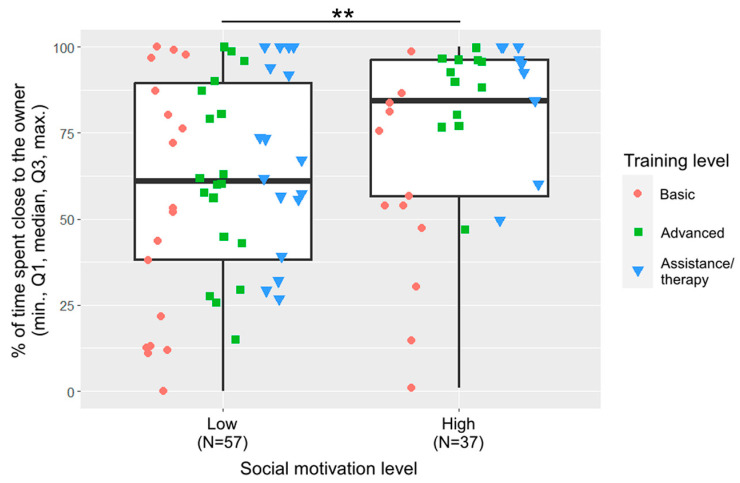
Percentage of time spent close to the owner by the two social motivation groups. (**: *p* < 0.01). The different shapes show the three training levels as individual data points within the social motivation groups.

Training level and social motivation were included in the final model of closeness (Figure 4). Dogs with basic training spent less time close to the owner than dogs with advanced training (β ± SE  = −41.3 ± 15.9, *p* = 0.012) or assistance/therapy dogs (β ± SE  = −51.5 ± 17.0, *p* = 0.010). Dogs with low social motivation spent less time close to the owner than dogs with high social motivation (β ± SE  = −38.7 ± 13.8, *p* = 0.006).

**Figure 5:**
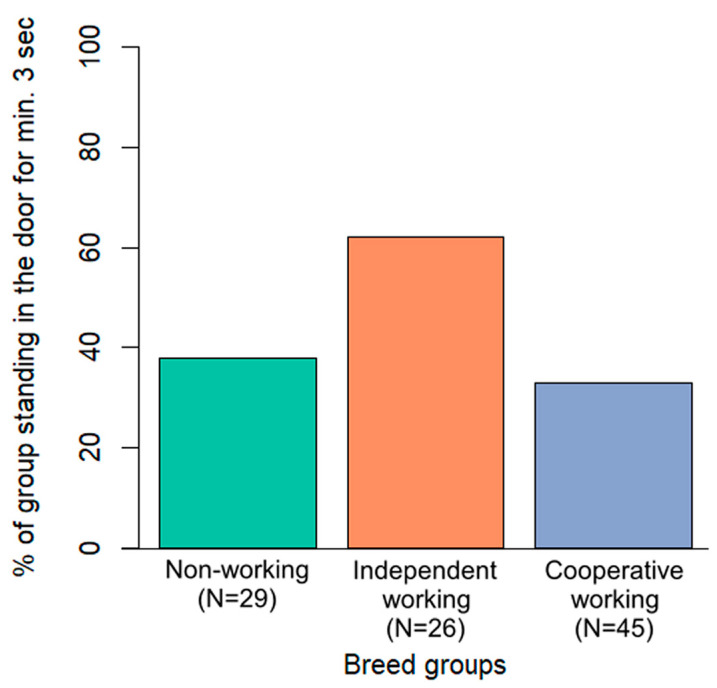
Percentage of dogs in the three breed groups standing at the door leading to the target room for at least three consecutive seconds during the test phase, while the owner was not nearby.

Only breed group was included in the final model of the door variable. Dogs from the independent working breed group appeared more likely to remain near the door leading to the target room while the owner was not nearby, compared to the other two breed groups (Figure 5). However, after the post hoc test, this difference was not significant (*p* > 0.07 in all cases).

**Figure 6:**
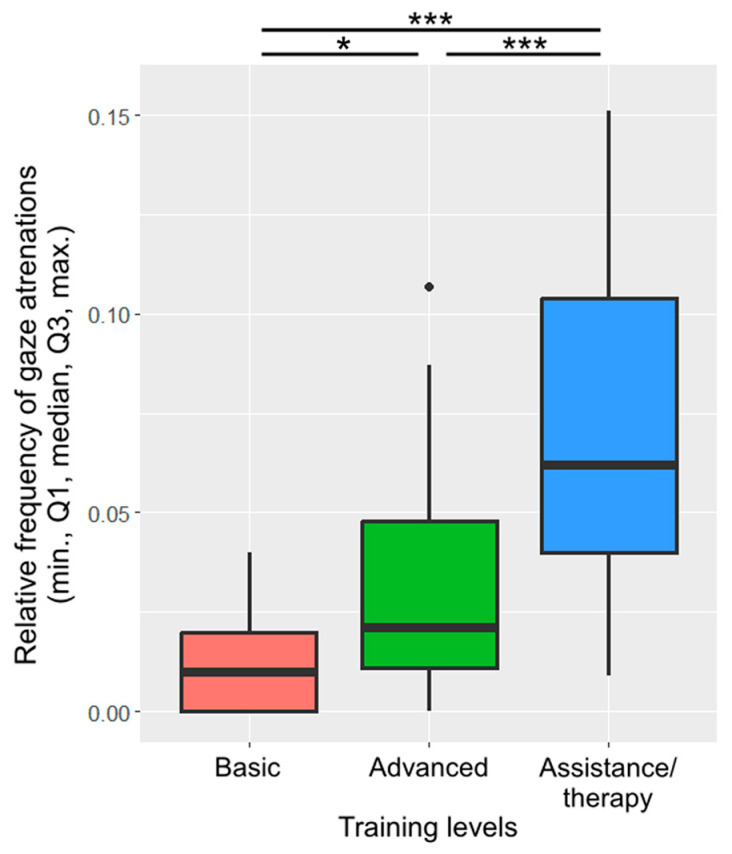
The relative frequency of gaze alternations between the owner and the key across the different training levels. (*: *p* < 0.05; ***: *p* < 0.001).

Training level was included in the final model of gaze alternation. Assistance/therapy dogs alternated their gaze more frequently than dogs with basic training (β ± SE = −0.06 ± 0.01, *p* < 0.001; Figure 6) or advanced training (β ± SE = −0.04 ± 0.01, *p* < 0.001). Dogs with advanced training also alternated their gaze more frequently than dogs with basic training (β ± SE = −0.02 ± 0.01, *p* = 0.022).

None of our model terms reached a model-averaged importance of at least 0.8 regarding the occurrence of vocalisation.

Only training level was included in the final model of approaching the key at least once. Assistance/therapy dogs were more likely to approach the key at least once during the problem situation than dogs with basic training (β ± SE  = −1.70 ± 0.71, *p* = 0.041) or advanced training (β ± SE  = −1.55± 0.71, *p* = 0.041).

**Figure 7:**
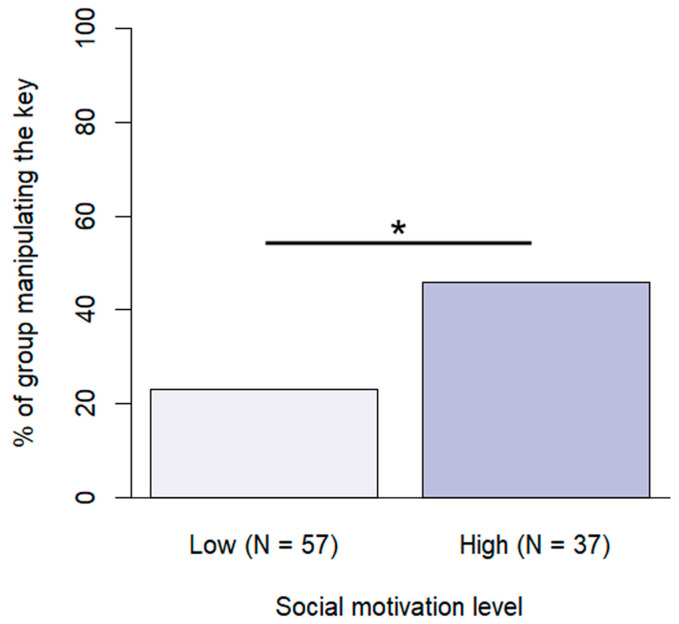
Percentage of subjects manipulating the key at the different social motivation levels. (*: *p* < 0.05).

Training level and social motivation were included in the final model of manipulating the key (Figure 7). Assistance/therapy dogs were more likely to manipulate the key at least once during the problem situation than dogs with basic training (β ± SE  = −2.18 ± 0.67, *p* = 0.003) or advanced training (β ± SE  = −1.26 ± 0.57, *p* = 0.042). Dogs with high social motivation were also more likely to manipulate the key (β ± SE  = −1.33 ± 0.51, *p* = 0.009).

## 4. Discussion

The aim of this study was to investigate how (I) breed function, (II) training experience, and (III) social motivation affect dogs’ spontaneous behaviour in a cooperative task with the owner as a partner. Differences were expected in all three steps of the process: (1) paying attention to the partner, (2) understanding the problem situation, and (3) willingness to help the partner in achieving a common goal.

Interestingly, in the context of this study, breed function had little to no effect on dogs’ behaviour. In previous studies, cooperative breeds proved to be particularly successful in utilising certain human communicational cues (e.g., pointing: [23]; latency to form eye contact: [24]), which likely enhances the success of cooperative interactions. However, with one exception, the breed groups we created had no effect on any of the measured variables in our study. Independent working breeds showed a tendency to stand in the door leading to the target room during the test phase. This suggests a preference for solving the problem on their own rather than relying on the owner, or possibly a higher level of food motivation. Contrary to our expectations, non-working dogs were more similar to the cooperative breed group than to the independent breed group. These findings align with the results of [33], who found inconsistent differences between breed groups, while training experience had a strong impact on dogs’ problem-solving behaviour in a manipulation and a detour task. Of course, the lack of significant effect of breed function in this specific task does not negate the potential influence of breed on dogs’ behaviour in other cooperative contexts.

A variety of studies have now established that training does not always have a clear and distinct effect on dogs’ behaviour. Dogs that received special training for the use of human visual communicational signals (i.e., agility training) were expected but not proved to be better at relying on a human’s distal pointing gestures [7]. These results somewhat contrast with those of [25], who reported that assistance and therapy dogs outperformed untrained dogs and dogs trained for recreational purposes in a problem-solving task, in which dogs had to manipulate an apparatus. Marshall-Pescini et al. [34] also reported that in a problem-solving task, search and rescue dogs acted more independently of humans than untrained and agility dogs (in the solvable trials). In the unsolvable trials, rescue dogs alternated their gaze between the manipulated apparatus and humans more frequently than untrained dogs, with agility dogs falling in the middle.

Our results are consistent with the latter, as even in a relatively simple problem situation that did not require any pre-training, training experience seemed to significantly influence dogs’ spontaneous attention and cooperativity-related behaviours. One plausible explanation for these results could be that highly trained dogs are likely more adept at recognising and solving (novel) tasks and may be more attuned to their owner’s behaviour. This effect could have been further strengthened by owners not being allowed to communicate with the dog during the test.

However, it is challenging to determine which factor is most important in this regard: the nature of the training itself, the quality time spent with the owner, or both. In human children, a higher parental connectedness (including mutual parent–child positive engagement, reciprocity, intimacy, and happy emotional tone) was positively related to kindergarteners’ prosocial tendencies [35].

It is important to acknowledge that although training experience was strongly associated with almost every behavioural variable in our study, it does not solely explain everything behind dogs’ cooperativity. The majority of our subjects showed quite high levels of attentiveness regardless of their training background (Figure 2), and not only did highly trained dogs manipulate the key, but some relatively untrained dogs also did so.

Of course, one specific context is not enough to identify all of the factors that could influence dogs’ spontaneous cooperativity. We aimed to find a context that could be easily solved and did not require pre-training. However, since the solution of the problem involved object manipulation, it is possible that highly trained individuals and breeds specifically selected for fetching tasks had an advantage in the task. Another limitation of this study is that despite our best efforts to standardise the experimental setup, the convincingness of the owners’ acting must have varied, which may have influenced dogs’ behaviour. Moreover, it cannot be excluded that dogs’ cooperative behaviours were influenced by selfish motives (expecting food), even if they did not receive food rewards after every single warm-up trial. While we did not condition dogs for solving this specific task, and the owner did not give any order or used known words, dogs are often rewarded with food in their everyday lives. This regular exposure may lead to dogs having expectations after receiving a food reward in a specific situation, even if it occurred only on a few occasions, at specific locations and at specific times. In addition, although owners were asked to refrain from handling the keychain after they touched the cheese, they still had to touch the key itself. As such, we could not completely prevent the smell/taste of cheese smearing on the object, potentially influencing the dogs’ behaviour. However, this could have affected all of our subjects equally; thus, it should not pose a significant effect on the interpretation of our results.

Our study revealed that dogs with high social motivation showed increased attention and cooperativity. Similarly to the dogs in our sample, social rewards are highly valued by children: certain age groups find them more rewarding than material rewards [36]. In fact, the use of material rewards can even undermine children’s helping behaviour [37], particularly in cases where mothers feel positively about using rewards [38]. As [36] suggested, this phenomenon could be caused by these children associating their helpful actions with external rewards instead of internal motivations, which could also explain our findings in dogs.

In addition to the similarities observed between dogs and children, it has been suggested that the study of dog–human cooperation can also contribute to the development of different research fields, such as social robotics [39]. According to [11], robots can be regarded as separate species, and dog–human interactions are probably the best available models for assistance robot–human interactions. Using a species that is socially competent and efficient in cooperating with humans, although behaviourally and morphologically different, could be a suitable decision for this purpose. Hence, we propose that, when designing robots that work in close collaboration with humans, some relevant features of the cooperative behaviours of dogs could be taken into consideration to make robot–human interactions more natural. Our findings suggest that a highly cooperative robot should spend a significant amount of time monitoring the partner’s actions (orienting, staying close; as also proposed by [40]), as increased attention and ‘readiness’ to engage in interactions with the partner appear to spontaneously lead to a more successful and natural cooperation.

Nevertheless, as previously stated, dogs’ cooperativity holds inherent intrigue and importance beyond its practical implications. Future studies might delve into the evolutionary background of these behaviours by conducting direct comparisons between dogs and their closest living relatives, wolves, or between family dogs and companion cats, which are typically kept under comparable conditions.

## 5. Conclusions

In summary, our results highlight the significance of training experience and social motivation in dogs’ spontaneous attention towards the owner’s actions in non-training-related problem situations, and their spontaneous cooperativity in such contexts. To ensure the validity of future studies, it appears necessary to either carefully balance the sample for these factors, or exclude subjects with specific training experiences, such as assistance dogs.

## Figures and Tables

**Table 1:** Basic information regarding subjects’ breed groups, training background, age, and sex, with the number of neutered individuals in brackets.

		Breed Groups		
		Non-Working Dogs (N = 29)	Independent Working Breeds (N = 26)	Cooperative Working Breeds (N = 45)
Training level	Basic	9	9	15
	Advanced	16	11	13
	Assistance/therapy	4	6	17 *
Sex	Male	19 (17 neut.)	13 (10 neut.)	27 (22 neut.)
	Female	10 (9 neut.)	13 (11 neut.)	18 (16 neut.)
Mean age ± SD (years)		5.2 ± 3.2	5.7 ± 3.9	5.6 ± 2.9

* The assistance/therapy dog group could not be balanced further, because typically cooperative breeds are chosen for this purpose.

**Table 2:** List of the coded behaviours and their definitions. (* paddling was excluded from the analyses).

Component	Coded Behaviour	Definition	Measure	Period When Coded
Attentiveness	Orienting at owner	Orienting towards the owner.	Time percentage	Warm–up phase, Testing phase
	Closeness to owner	Being near the owner (in reaching distance).	Time percentage	Testing phase
	Door	Approaching the door of the target room (within 1 m) when the owner is not in reaching distance, and remaining there for at least 3 consecutive seconds.	Yes/no	Testing phase
	Gaze alternation	Gazing at the owner is followed directly by a gaze at the key or vice versa within 2 s.	Frequency	Testing phase
Distress response	Vocalisation	Any kind of vocalisation (e.g., barking, whining).	Yes/no	Testing phase
	Paddling *	The dog shifts its body weight quickly from one leg to another, moving in a scuffled, awkward way.	Frequency	Testing phase
Cooperativity	Approaching the key	Orienting towards the key while the dog’s snout is max. 20 cm away from it.	Yes/no	Testing phase
	Manipulating the key	Pawing or oral manipulation of the key, or moving the key with the snout.	Yes/no	Testing phase

## Data Availability

The data presented in this study are available on request from the corresponding author.

## Supplementary Materials

The following supporting information can be downloaded at: https://www.mdpi.com/article/10.3390/ani13142390/s1, Section S1: Questionnaire used to assess dogs’ training experience, fetching experience, and social motivation level; Section S2: Detailed description of the statistical models; Table S1: Detailed information on sample demographics.
