# Supplementary material for: Factors Contributing to Successful Spontaneous Dog–Human Cooperation
Source: Animals (Basel). 2023 Jul 23;13(14):2390. doi: 10.3390/ani13142390 (PMC10376063; doi:10.3390/ani13142390)
Supplement: Supplementary file 1 [file animals-13-02390-s001.zip › animals-2447782-supplementary.pdf]

Supplementary material to:
Factors Contributing to Successful Spontaneous Dog–Human Cooperation

Melitta Csepregi[1,2,3,*] and Márta Gácsi[1,2]

[1]ELKH–ELTE Comparative Ethology Research Group, Budapest, Hungary

[2]Department of Ethology, Eötvös Loránd University, Budapest, Hungary

[3]Doctoral School of Biology, Institute of Biology, Eötvös Loránd University, Budapest, Hungary

*Correspondence: melittacsepregi@gmail.com

Section S1

The following questionnaire was used to assess dogs' training experience, fetching experience and social motivation level. Bulleted points indicate available categories provided for each question.

1. Owner's name
2. Email address, used only for the communication necessary for the tests and is not passed on to third parties.

1. The dog's name
2. The dog's sex
    - Male
    - Female
3. Is the dog neutered?
    - Yes
    - No
4. The dog's date of birth in YYYY.MM.DD format. If you do not know your dog's exact birth date, please, enter the following numbers: ″1212.12.12.″ and go to the next question where you can enter the estimated age of the dog.
5. Your dog's estimated date of birth. If you do not know exactly when your dog was born, please estimate its age.
6. The dog's breed

7. What type of training has your dog been involved in for an extended period of time? (In dog school or through other organised training.)
    - My dog has never participated in any kind of organised training
    - I trained my dog myself
    - Basic obedience training
    - Advanced obedience training
    - Agility
    - Defence
    - Rescuing
    - Mantrailing
    - Hunting
    - My dog is a therapy dog
    - My dog is an assistance dog
8. How well trained is your dog in fetching tasks?
    - Not trained at all (score 0)
    - I throw its toys when we play and it usually brings them back (score 1)
    - On request, my dog fetches simple objects (not just toys/sticks/dummies), even if it’s just down on the floor and is not thrown (score 2)
    - On request, my dog fetches almost any object, can hold it in its mouth and put it down when I ask it to do so (score 3)
    - My dog can place any object in/on other objects, and can be sent to others with objects in its mouth (score 4)
    - My dog picks up dropped objects spontaneously by itself (score 5)
9. Can your dog be easily motivated with verbal praise, petting, petting? (Without using treats.)
    - Yes, over a prolonged period of time
    - Yes, but only for a short period of time
    - No

Section S2

Detailed description of the best models, and the final models reported in the results for each behavioural variable. Since the AICc values of our models were close to each other, after using

the glmulti function, we plotted the relative importance of model terms (i.e., the overall support for each variable across all models). The selection of the final model for each behavioral variable was made based on the following criteria: 1) the model had one of the lowest AICc values, and 2) its terms reached 0.8 model-averaged importance or higher.

Orienting at owner

Best models (within 2 IC units)

Model	AICc	Weights
orient ~ 1 + training_lvl	1944.07	0.448
orient ~ 1 + training_lvl + social_motivation	1945.40	0.230

Final formula = orient ~ training_lvl

	β	Std. Error	t value	Pr(>|t|)
(Intercept)	17167	1287	13.336	<0.001
training_lvl_advanced	2255	1781	1.266	0.209
training_lvl_assistance	6017	1903	3.162	0.002

Closeness to owner

Best models (within 2 IC units)

Model	AICc	Weights
close ~ 1 + training_lvl + social_motivation	1058.22	0.259
close ~ 1 + breed_group + training_lvl + social_motivation	1058.37	0.240
close ~ 1 + training_lvl + social_motivation + training_lvl:social_motivation	1059.28	0.152
close ~ 1 + breed_group + training_lvl + social_motivation + training_lvl:social_motivation	1059.61	0.129

Final formula = close ~ training_lvl + social_motivation

	β	Std. Error	t value	Pr(>|t|)
(Intercept)	101.82	12.98	7.864	<0.001
training_lvl_advanced	41.33	15.93	2.594	0.011
training_lvl_assistance	51.47	17.02	3.025	0.003
social_motivation_high	38.73	13.76	2.815	0.006

Door

Best models (within 2 IC units)

Model	AICc	Weights
door ~ 1 + breed_group + training_lvl + social_motivation + social_motivation:breed_group	125.61	0.241
door ~ 1 + breed_group + training_lvl	126.70	0.139
door ~ 1 + training_lvl	127.50	0.094
door ~ 1 + breed_group	127.52	0.092

Final formula = door ~ breed_group

	β	Std. Error	z value	Pr(>|z|)
(Intercept)	-0.49	0.38	-1.287	0.198
breed_group_independent	0.93	0.57	1.629	0.043
breed_group_cooperative	-0.31	0.51	-0.610	0.323

Gaze alternation

Best models (within 2 IC units)

Model	AICc	Weights
gaze ~ 1 + training_lvl	-53.20	0.538
gaze ~ 1 + training_lvl + social_motivation	-51.75	0.262

Final formula = gaze ~ training_lvl

	β	Std. Error	t value	Pr(>|t|)
(Intercept)	0.01	0.01	2.329	0.022
training_lvl_advanced	0.02	0.01	2.336	0.022
training_lvl_assistance	0.06	0.01	7.511	<0.001

Vocalisation

Best models (within 2 IC units)

Model	AICc	Weights
vocalisation ~ 1 + training_lvl	97.72	0.489
vocalisation ~ 1	99.35	0.216
vocalisation ~ 1 + training_lvl + social_motivation	99.68	0.184

Final formula = vocalisation ~ training_lvl

	β	Std. Error	z value	Pr(>|z|)
(Intercept)	-1.27	0.43	-2.977	0.003
training_lvl_advanced	0.49	0.56	0.877	0.380
training_lvl_assistance	-1.25	0.85	-1.473	0.141

Approaching the key

Best models (within 2 IC units)

Model	AICc	Weights
approach ~ 1 + breed_group + training_lvl + social_motivation	111.61	0.300
approach ~ 1 + breed_group + training_lvl	112.83	0.163
approach ~ 1 + training_lvl + social_motivation	113.33	0.110
approach ~ 1 + training_lvl	113.51	0.070

Final formula = approach ~ training_lvl

	β	Std. Error	z value	Pr(>|z|)
(Intercept)	0.38	0.36	1.054	0.292
training_lvl_advanced	0.15	0.50	0.292	0.770
training_lvl_assistance	1.70	0.71	2.393	0.017

Manipulating the key

Best models (within 2 IC units)

Model	AICc	Weights
manipulate ~ 1 + training_lvl + social_motivation + training_lvl:social_motivation	106.99	0.495
manipulate ~ 1 + training_lvl + social_motivation	107.91	0.313

Final formula = manipulate ~ training_lvl + social_motivation

	β	Std. Error	z value	Pr(>|z|)
(Intercept)	-2.42	0.60	-4.00	<0.001
training_lvl_advanced	0.92	0.64	1.44	0.149
training_lvl_assistance	2.18	0.67	3.264	0.001
social_motivation_high	1.33	0.51	2.605	0.009

Supplementary table S1. Detailed information on sample demographics.

ID	Sex	Neutered status	Age (years)	Breed	Breed group	Training level	Fetching experience	Social motivation
1	Female	Neutered	9.76	golden retriever	Cooperative	Assistance/therapy	5	Low
2	Male	Neutered	4.93	Tervueren	Cooperative	Assistance/therapy	5	High
3	Female	Neutered	1.15	German shepherd	Cooperative	Advanced	2	Low
4	Male	Neutered	11.00	mongrel	Non-working	Advanced	2	Low
5	Male	Neutered	N/A	Jack Russell terrier	Independent	Advanced	N/A	N/A
6	Female	Neutered	N/A	Jack Russell terrier	Independent	Advanced	N/A	N/A
7	Male	Intact	8.40	German shepherd	Cooperative	Advanced	3	Low
8	Female	Neutered	8.68	border collie	Cooperative	Basic	2	Low
9	Female	Neutered	8.04	Malinois – German shepherd mix	Cooperative	Advanced	3	High
10	Female	Intact	N/A	border collie	Cooperative	Advanced	N/A	N/A

11	Male	Neutered	N/A	border collie	Cooperative	Advanced	N/A	N/A
12	Female	Neutered	3.79	Hungarian vizsla	Cooperative	Basic	2	High
13	Male	Neutered	2.78	German shepherd	Cooperative	Advanced	2	High
14	Female	Neutered	8.92	mongrel	Non–working	Advanced	3	Low
15	Male	Neutered	6.67	Labrador retriever	Cooperative	Basic	0	Low
16	Female	Neutered	2.05	Malinois	Cooperative	Basic	2	High
17	Male	Intact	2.86	Ibizan hound (wire haired)	Independent	Assistance/therapy	4	Low
18	Male	Neutered	8.10	wire haired dachshund	Independent	Assistance/therapy	2	Low
19	Male	Neutered	14.54	cairn terrier	Independent	Assistance/therapy	4	Low
20	Male	Neutered	10.74	mongrel	Non–working	Advanced	3	High
21	Male	Neutered	4.47	golden retriever	Cooperative	Assistance/therapy	4	High
22	Male	Neutered	10.07	mongrel	Non–working	Basic	0	Low

23	Female	Intact	2.95	basset hound	Independent	Basic	0	Low
24	Male	Neutered	3.00	basset hound	Independent	Basic	0	Low
25	Female	Neutered	13.16	beagle	Independent	Assistance/therapy	3	Low
26	Male	Neutered	8.51	standard poodle	Cooperative	Assistance/therapy	3	High
27	Female	Neutered	11.28	puli	Cooperative	Assistance/therapy	4	Low
28	Male	Intact	8.66	Malinois	Cooperative	Assistance/therapy	5	High
29	Male	Neutered	8.95	Labrador retriever	Cooperative	Assistance/therapy	4	Low
30	Female	Neutered	3.24	Labrador retriever	Cooperative	Assistance/therapy	4	High
31	Female	Intact	1.85	Malinois	Cooperative	Assistance/therapy	3	Low
32	Male	Neutered	5.70	puli	Cooperative	Assistance/therapy	5	Low
33	Male	Neutered	2.96	English cocker spaniel	Cooperative	Basic	3	High
34	Male	Neutered	11.59	beagle	Independent	Advanced	0	Low

35	Male	Intact	2.01	beagle	Independent	Advanced	2	Low
36	Male	Neutered	9.60	Jack Russell terrier	Independent	Advanced	2	Low
37	Male	Neutered	8.30	Parson Russell terrier	Independent	Basic	2	Low
38	Female	Neutered	1.94	beagle	Independent	Basic	1	Low
39	Female	Neutered	9.66	Hungarian vizsla	Cooperative	Advanced	3	Low
40	Male	Neutered	8.63	standard poodle	Cooperative	Basic	2	Low
41	Male	Neutered	4.85	mongrel	Non–working	Advanced	2	High
42	Male	Neutered	3.00	mongrel	Non–working	Advanced	1	High
43	Female	Neutered	2.99	Labrador retriever	Cooperative	Advanced	0	Low
44	Male	Neutered	3.39	mongrel	Non–working	Assistance/therapy	2	High
45	Male	Neutered	11.00	mongrel	Non–working	Assistance/therapy	0	Low
46	Male	Neutered	4.00	chihuahua	Non–working	Advanced	1	Low

47	Male	Neutered	10.83	English setter	Cooperative	Basic	1	Low
48	Male	Neutered	4.58	Pembroke Welsh Corgi	Cooperative	Basic	0	Low
49	Male	Neutered	1.29	Pembroke Welsh Corgi	Cooperative	Basic	2	High
50	Female	Neutered	4.23	mongrel	Non–working	Advanced	5	High
51	Male	Neutered	11.00	mongrel	Non–working	Basic	0	Low
52	Female	Neutered	1.58	mongrel	Non–working	Advanced	1	Low
53	Female	Neutered	7.05	saluki	Independent	Assistance/therapy	0	Low
54	Male	Neutered	5.93	mongrel	Non–working	Assistance/therapy	1	High
55	Male	Neutered	1.88	dachshund	Independent	Advanced	1	Low
56	Male	Neutered	6.25	Pembroke Welsh Corgi	Cooperative	Basic	0	Low
57	Male	Intact	3.29	pumi	Cooperative	Basic	0	Low
58	Female	Neutered	8.09	German shepherd	Cooperative	Basic	1	High

59	Male	Intact	3.61	pumi	Cooperative	Basic	1	High
60	Female	Neutered	4.19	miniature dachshund	Independent	Advanced	0	High
62	Male	Neutered	5.13	border collie	Cooperative	Advanced	2	Low
63	Male	Neutered	1.27	English bulldog	Non–working	Basic	0	High
64	Female	Neutered	3.00	mongrel	Non–working	Advanced	1	Low
65	Male	Neutered	4.00	borzoi	Independent	Basic	N/A	N/A
66	Male	Neutered	2.00	mongrel	Non–working	Basic	2	Low
67	Female	Neutered	5.08	mongrel	Non–working	Basic	0	High
68	Female	Neutered	1.87	Jack Russell terrier	Independent	Basic	0	High
69	Male	Neutered	4.03	shiba inu	Non–working	Advanced	2	Low
70	Female	Neutered	5.08	Transylvanian hound	Independent	Advanced	0	High
71	Male	Neutered	4.00	mongrel	Non–working	Basic	0	High

73	Male	Neutered	2.26	Pembroke Welsh Corgi	Cooperative	Basic	1	High
74	Male	Neutered	1.82	Bichon Havanese	Non–working	Assistance/therapy	3	Low
75	Male	Neutered	6.01	American Staffordshire terrier	Non–working	Basic	2	Low
76	Female	Intact	1.15	Irish soft coated wheaten terrier	Independent	Basic	0	Low
77	Female	Neutered	8.00	wire haired dachshund	Independent	Basic	1	High
78	Female	Neutered	8.00	wire haired dachshund	Independent	Advanced	1	High
79	Male	Neutered	3.00	German pointer	Cooperative	Basic	0	Low
80	Male	Intact	2.29	dogo argentino	Non–working	Basic	0	Low
81	Female	Neutered	5.98	miniature bull terrier	Non–working	Advanced	1	Low
82	Male	Neutered	9.00	mudi	Cooperative	Advanced	N/A	N/A
83	Male	Neutered	5.45	labradoodle	Cooperative	Assistance/therapy	4	High
84	Male	Neutered	9.86	English cocker spaniel	Cooperative	Advanced	2	High

86	Female	Neutered	1.81	Welsh terrier	Independent	Advanced	0	Low
87	Male	Intact	6.68	Bichon Havanese	Non–working	Advanced	1	Low
88	Male	Neutered	6.00	mongrel	Non–working	Advanced	4	Low
89	Female	Neutered	2.50	border collie	Cooperative	Advanced	4	High
90	Male	Intact	3.47	Jack Russell terrier	Independent	Advanced	2	High
91	Male	Neutered	8.28	West Siberian laika	Independent	Basic	0	High
92	Female	Intact	1.08	shiba inu	Non–working	Basic	1	Low
93	Female	Neutered	2.00	mongrel	Non–working	Advanced	1	Low
94	Male	Intact	8.62	Australian shepherd	Cooperative	Advanced	3	Low
95	Female	Neutered	3.00	mongrel	Non–working	Advanced	4	High
96	Female	Neutered	7.00	mongrel	Non–working	Advanced	2	Low
97	Female	Neutered	3.41	border collie	Cooperative	Assistance/therapy	5	High

98	Female	Neutered	4.27	Labrador retriever	Cooperative	Assistance/therapy	4	Low
99	Male	Neutered	3.60	Portuguese water dog	Cooperative	Assistance/therapy	1	Low
100	Female	Neutered	6.42	Portuguese water dog	Cooperative	Assistance/therapy	4	Low
101	Female	Neutered	2.43	Australian shepherd	Cooperative	Assistance/therapy	4	High
102	Male	Neutered	3.37	border collie	Cooperative	Assistance/therapy	3	Low
103	Female	Neutered	3.37	wire haired dachshund	Independent	Assistance/therapy	1	Low
